# Coconut Rhinoceros Beetle in Samoa: Review of a Century-Old Invasion and Prospects for Control in a Changing Future

**DOI:** 10.3390/insects13050487

**Published:** 2022-05-23

**Authors:** Sulav Paudel, Sean D. G. Marshall, Nicola K. Richards, George Hazelman, Pueata Tanielu, Trevor A. Jackson

**Affiliations:** 1 AgResearch Limited, 1365 Springs Road, Lincoln 7674, Private Bag 4749, Christchurch 8140, New Zealand; sulav.paudel@agresearch.co.nz (S.P.); sean.marshall@agresearch.co.nz (S.D.G.M.); nicky.richards@agresearch.co.nz (N.K.R.); 2 Ministry of Agriculture and Fisheries, Tui Atua Tupua Tamasese Efi Building, Apia 38360, Samoa; george.hazelman@maf.gov.ws (G.H.); pueata.tanielu@maf.gov.ws (P.T.)

**Keywords:** coconut rhinoceros beetle, *Oryctes rhinoceros*, Samoa, Oryctes rhinoceros nudivirus, integrated pest management, biological control

## Abstract

**Simple Summary:**

Coconut rhinoceros beetle (CRB) is one of the major pests of coconut and oil palms in the Asia-Pacific region. Since its accidental introduction in Samoa in 1909, the invasive CRB has spread to several countries and territories within the Pacific and Indian Oceans, severely damaging coconut palms and affecting peoples’ livelihoods. In the 100 years since CRB established on Samoa, it remains the primary pest on the island with periods of heavy damage when integrated pest management (IPM) breaks down. The Samoan case is an excellent example of implementing biocontrol and IPM in a dynamic Pacific environment. As society and the economics of production in Samoa have changed, the level of control has varied, with recent concern about surges of the pest. The review synthesizes historical lessons and provide recommendations on how to protect coconut palms in the changing environment of Samoa which are also applicable for protection of palms in the wider Asia/Pacific region.

**Abstract:**

It is now more than 100 years since the coconut rhinoceros beetle (CRB: *Oryctes rhinoceros* L.) was first detected in the Pacific Island state of Samoa. The exotic pest from Asia became the principal pest of coconut palms in Samoa and, from this first point of invasion, spread to several surrounding countries in the South-West Pacific Ocean. An intensive control operation was initiated, but the beetle could not be eliminated. Various pest management strategies were attempted but had limited success until the introduction of a biological control agent (BCA), Oryctes rhinoceros nudivirus (OrNV), during the late 1960s and early 1970s. The biocontrol release was very successful and became the prime example of “classical biological control” of an insect pest by a virus. Changing economic and social conditions in Samoa and other islands of the Pacific require a re-evaluation of the threat of CRB to coconut production to suggest how the IPM system may be modified to meet future needs. Therefore, it is timely to review the history of CRB in Samoa and summarize experiences in development of an integrated pest management (IPM) system limiting the impact of the pest. We also present results from a recent study conducted in 2020 on the island of Upolu to define the current status of the CRB population and its BCA, OrNV. The lessons from Samoa, with its long history of containment and management of CRB, are applicable to more recent invasion sites. Recommendations are provided to modify the IPM programme to enhance the sustainable control of CRB and support the ongoing coconut replantation program promoted by the Samoan government.

## 1. Introduction

**Figure 1:**
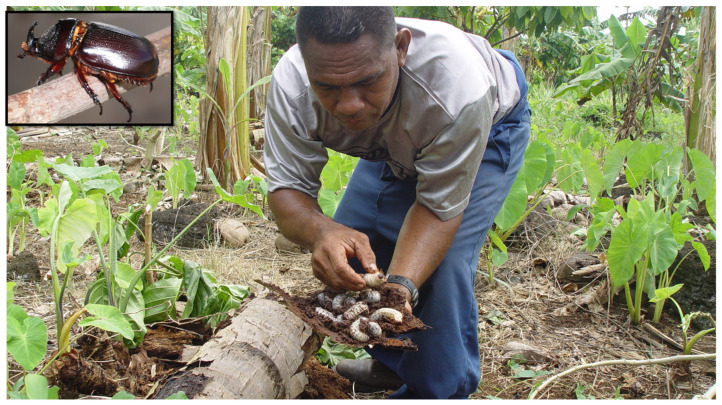
Collection of CRB (*Oryctes rhinoceros*) larvae from a rotting palm trunk in Samoa. Insert (top left) shows the adult beetle.

The coconut rhinoceros beetle (CRB: *Oryctes rhinoceros* L.) is endemic to South/South East Asia, where it has a close association with palm trees for feeding and breeding and can be a pest of cultivated palms when beetle numbers are high [1]. CRB has become a major pest of coconut (*Cocos nucifera*) and oil palm (*Elaeis guineensis*) through the invasion of islands in the Pacific and Indian Oceans [1]. It has also been reported to damage more than 30 different genera of plants, including sugarcane, pineapple, pandanus, banana, taro, cycads, and agaves. Adult beetles cause damage by mining into the emerging spear and feeding on sap from the soft developing leaf tissues. In cases of severe attack, beetles will damage the meristem and kill the palm within a few months. As the palm decays, the rotting plant material is an excellent breeding source for females and larval development. CRB larvae feed and develop on the rotting stems, pupating and emerging as adults to continue the damage cycle (Figure 1). In the absence of effective control measures, the beetle has a devastating effect and can cause a palm mortality of 50% or more [2,3]. Although the direct transmission of disease by CRB is not reported, beetle damage to the palms increases the risk of secondary infections and infestations from fungi, bacteria, viruses, and weevils [1].

CRB was first reported in the Pacific islands from Upolu, Samoa in 1910, apparently after accidental introduction in planting material shipped from Ceylon (Sri Lanka) [4]. The beetle population rapidly increased, causing severe damage to coconut palms, and spread throughout the Samoan islands, at that time under German and United States administration [4,5]. Although the beetle is a strong flyer, long distances between islands limited their spread. Therefore, movement between islands during that time was assumed to be mostly through accidental transport on local shipping [4]. In 1912, the beetle was recorded from Tutuila, American Samoa [2].

The first record of CRB beyond the Samoan islands was from the Tongan island of Niuatopu-tapu (Keppel Island) in 1922, but a thorough and intensive clean-up operation on this island led to their eradication by 1929 [2,6]. The beetle was reported in Wallis Island in 1931, where it established itself, and the population has persisted [2]. The range of expansion of CRB during that period (1909–1940) was limited due to the remoteness of the islands, infrequent inter-island shipping, and the application of quarantine measures.

Increased shipping movement during WWII and the post-war years accelerated CRB spread [2]. The beetle invaded Tonga and Fiji during the 1950s and, once within these countries, spread throughout the island groups [2,7]. At the same time, a second wave of invasion occurred in the northern offshore islands of Papua New Guinea. A new variant of the beetle (CRB-G) has been detected, more recently (starting from 2007), in several Pacific Island countries and territories (PICTs) [8].

In the islands of the Pacific, CRB populations grew rapidly in the absence of natural enemies, causing damage to coconut palms, which were essential as food for the local populations and the production of copra as an export crop. The residing colonial governments engaged foreign experts, and, in time, a management system was developed, relying heavily on crop sanitation (removal of breeding materials, trapping, and quarantine), involving a large workforce of local labour [2,5,9,10]. In the 1960s, an international project funded through United Nations Development Programme (UNDP) and administered through the South Pacific Commission (SPC) was established to find a solution to the CRB problem, which was, by then, spreading widely through the PICTs [11].

Biological control, using natural enemies of the pest, was the favoured option to control the spreading pest. Several biological control agents (BCAs), including bacteria, predators, and parasitoids, were introduced into the infested PICTs during the 1950s and 1960s, but the anticipated success was never achieved [12,13,14]. In 1963, a German scientist (Dr. Alois Huger) was hired by the SPC to find an effective pathogen against CRB [15]. Based on the vigor of the invasive CRB populations, Huger was convinced that CRB had “escaped” the control of natural enemies from its endemic zone and initiated a search for BCAs in the endemic region of Malaysia [15]. The search led to the discovery of larvae with a disease phenotype (stunting, glassy appearance, and a prolapsed abdomen) and, later, the identification of adults that had ceased feeding and were characterized by a swollen and milky midgut [16]. Diseased insect macerates were fed to healthy beetles, which produced similar symptoms. Using histology and electron microscopy, the causal agent was identified as a non-occluded virus, first described as *Rhabdionvirus oryctes* and, later, *Oryctes* baculovirus, until its current classification as Oryctes rhinoceros nudivirus (OrNV) [15,16].

The invasive beetle populations were successfully managed in Samoa after the introduction of an OrNV during the late 1960s in combination with sanitation activities [1,15]. Success of the virus in Samoa led to releases in other invaded areas of the PICTs (e.g., Fiji, Tonga, PNG) throughout the 1970s and 1980s. In all cases, infection was recorded in the target populations, and dramatic reductions in damage to palms were noted within 18–24 months of the virus release [17,18,19]. The spread of the beetle to uninfested islands ceased for the next 30 years, presumably due to the weakening of the pest population. In 2007, a new outbreak of CRB was reported from the island of Guam and, subsequently, from PNG, Hawai’i, and Solomon Islands, where the beetles have since caused extensive damage [8,20,21,22]. Mitochondrial DNA sequencing revealed that the outbreak was caused by a new variant of rhinoceros beetle, designated as CRB-G [8]. The amplification of DNA from beetle guts using OrNV-specific primers showed that OrNV was generally present at high incidence within the established populations of CRB but was generally absent from the newly invasive CRB-G populations [8]. Further genetic analysis showed that CRB populations can be divided into several clades (I, II, III and IV) that coincide with the endemic and invasive history of the beetle [8]. Clades II, III, and IV, while genetically distinct, were collectively named CRB-S and characterized biologically by susceptibility to the original strain of OrNV used to control the earlier wave of CRB invasion in the Pacific. CRB specimens collected from Samoa were determined to be clade III. The fourth clade (clade I), which included populations from the newly invaded regions (Guam, Papua New Guinea, Hawai’i, Solomon Islands) and from parts of the native range (Philippines, Indonesia), was collectively named CRB-G. This new wave of invasion is threatening the sustainability of the coconut industries in PICTs [8,21].

In this study, we review the characteristics of the CRB invasion of the Samoan islands (being the earliest in the Pacific), the responses to the invasion, and lessons learnt through its management. We incorporate results of new studies to characterise the current status of CRB population and BCAs in Samoa. Lastly, we discuss the threat CRB poses to the reviving coconut/copra industry across the Pacific region and provide recommendations for the improved control and containment of CRB in Samoa and the wider Pacific region.

### 1.1. History of CRB in Samoa and Its Management Strategies

#### 1.1.1. CRB Detection in Samoa

**Figure 2:**
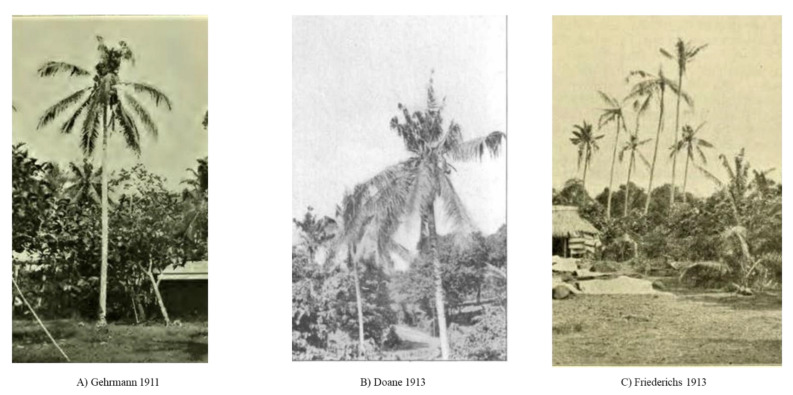
Photographs of damage recorded by early CRB researchers on Samoa. (**A**) Gerhmann 1911; (**B**) Doane 1913; (**C**) Friederichs 1913.

**Figure 3:**
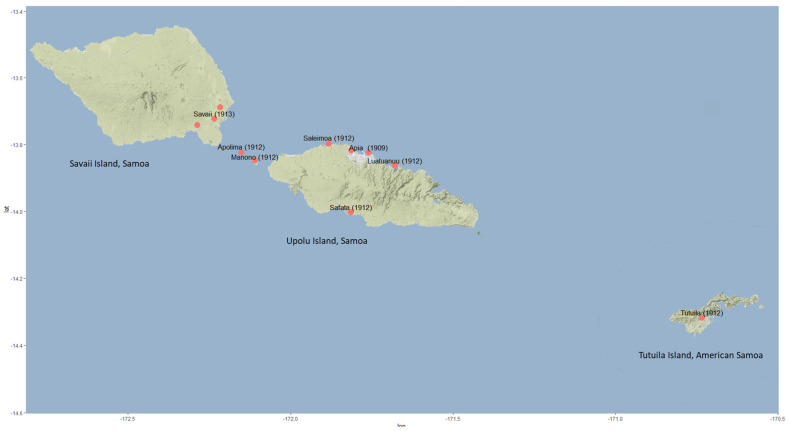
Spread of CRB (*Oryctes rhinoceros*) within the Samoan islands after its first confirmation in Apia, Samoa 1910. Longitude values are shown on the x-axis and the latitude values on the y-axis.

Coconut palm frond damage, characteristic of that produced by CRB, was first noted close to the Customs House, Apia in Nov 1910 and attributed to *O. rhinoceros* (initially identified as *O. boas*) [5]. Photographs of palms showed heavy damage to the upper fronds, indicative of recent but heavy infestation (Figure 2) [23]. Although the first signs of beetle damage were noted in Oct. 1910, it was recognized that the beetle had arrived in the country earlier (1909) [2]. The most likely source was on rubber plant (*Hevea brasiliensis*) “stumps” (cuttings), which were imported in bulk in 1908 into Samoa from Ceylon for the establishment of plantations [2,24]. The arrival of CRB and subsequent reports of heavy damage caused consternation in the region. Frank Jepson, the Government Entomologist, was dispatched from Suva, Fiji to evaluate the situation during a four-week visit in April–May 1912 [5]. The presence of CRB was confirmed along the north-west coast of Upolu and was found to be already widespread (Figure 3) [5]. Around 75% of the palms around the districts of Apia and Saleimoa showed signs of CRB damage. German entomologist Karl Friederichs arrived in Samoa in October 1912 and confirmed the establishment of the pest on the small islands of Manono and Apolima and the east coast of the island of Savaii [25]. Another expert, R.W. Doane from USA, visited during May–July 1913 and noted that hundreds of trees were being killed at the centre of the outbreak, and the beetle had continued to spread [4]. By the 1920s, all coconut-producing areas of Samoa were infested with the beetle.

#### 1.1.2. Initial Response and Government Ordinances

The initial response to the invasion was to kill the beetle on the affected palms or prevent attack. Tar, kerosine, detergent, and even sand were applied to protect palms from attack, and direct extraction of beetles with a hooked wire was used [4]. Beetle breeding sites were targeted with loose organic matter gathered, and dead palms were removed. It was noted that damage was limited in plantations under strong management with sanitation and beetle collection [25].

Trapping was also used and is described by Jepson [5]. Taking advantage of the beetles’ attraction to decaying organic matter for oviposition and larval development, traps were constructed by digging a hole 3–4 m^2^ to a depth of 75 cm. The pit was then filled with rotten coconut and banana stumps and covered with leaves. A surround was made of old coconut trunks and the pits filled to about 30 cm above ground level. The traps were opened every 6 weeks to 2 months, and the trapped CRB were collected and destroyed. Checking the content of such large traps was laborious; it took six men 2.5 h to complete each trap. Despite the effort involved, traps became common and were installed every 100 m along the roads and with one trap for every hundred trees in the plantations. The traps had a double benefit; the collection and destruction of beetles and the removal of organic matter, which becomes potential CRB breeding material. Friederichs designated the pits as “catch traps” and considered them indispensable in the fight against the beetle. He reported that in 1912/13 almost one million CRB larvae and other stages were captured in the traps and destroyed [25].

Alternatives to costly manual collection were sought. Chemicals were the first option tested. Jepson applied carbon bisulphide to traps as a fumigant and found it was 100% effective in killing CRB larvae [5]. Friedrichs had variable results from the use of carbon bisulphide and tested a further range of chemicals against CRB larvae in the pits, confirming the effect of fumigation, but considered that the availability and cost of suitable chemicals would limit their widespread application [25].

When checking samples collected from the field, Friederichs noted signs of a fungal disease in larvae [25]. He isolated a fungus and treated healthy larvae with fungal spores, establishing the same symptoms of infection, confirming Koch’s Postulates. The fungus caused the infection and death of treated insects and was identified as *Metarhizium anisopliae* [25]. The larval stage was most susceptible to the fungus, and death was followed by prolific sporulation, leaving the cadaver with a green coating of fungal spores. As the fungus-infected larvae were collected from the local plantations, it was postulated that the disease must have been present on the island prior to the arrival of CRB in another beetle host. Friederichs then had the idea that the fungal spores could be used to treat the beetle traps to kill the beetles and eliminate the high costs of regular searching and hand collection from the traps. Healthy CRB larvae were placed in containers with soil contaminated with spores of *M. anisopliae* and became infected, producing further spores. The freshly contaminated soil and cadavers were then added to the catch traps of organic matter under colonization by free living beetles. This method was found to be highly effective, often providing total control of the CRB larvae developing in the traps. Friederichs developed a system for planters to use for their own multiplication of the fungus and reported successful implementation of the method in plantation management. However, he recognized that the fungus had limited ability for auto-dispersion and recommended its application to the traps as part of an integrated pest management (IPM) system [25].

The first wave of entomologists (Jepson, Doane, and Friedrichs) assessing the CRB outbreak all commented that the absence of natural enemies for CRB on Samoa was the likely cause of the highly damaging outbreak. The presence of invertebrate natural enemies within the country was also investigated, but nothing significant was found [4]. The introduction of the hymenopteran *Scolia* spp. was suggested, which were known to be parasites of the Scarabaeidae in other tropical regions [5,25].

In the absence of effective BCAs, CRB management was enforced by strict regulations. A Government Ordinance was issued in Nov 1910, offering a reward for beetles collected (36 cents for every 20 beetles or 50 grubs) [26]. Coconut planting in new areas was stopped, with the mandatory clean-up of existing plantations. The use of coconut stems in construction was also prohibited. Despite spending GBP 2000 for the management program, satisfactory results were not achieved. Therefore, in Feb. 1912, rules of mandatory beetle collection/destruction and maintenance of clean plantations were enacted. All able-bodied men had to collect a quota of beetles each Wednesday and present them to the headman (pulenu’u). Vast numbers were collected. Jepson reports that 5.5 million larvae and 100,000 adult beetles were collected prior to February 1912 [5]. Friederichs later reported that ten million larvae and 250,000 beetles were collected and destroyed in the following year from April 1912, with 820,000 collected from the environs of Apia in a single month [25]. It was mentioned that the collection figures could be exaggerated through the mistaken identification of some insects but still represented massive numbers. Despite changes in Samoan administration, beetle collection continued as a key component in the CRB response. In 1915, a competition was held, and 1170 collectors competed for prizes and collected 19,309,923 larvae and 357,618 beetles [27].

The Beetle Ordinance of 1921 declared it was a duty of the “Occupiers of lands” to keep their lands clean of rotting trees and other CRB breeding materials [10]. Foreign laborers had to work for six hours on Monday mornings to collect beetles. Beetle numbers were recorded and destroyed by employers. Every Samoan male capable of searching had to collect a set quota of beetles and deliver them to the village pulenu’u. Failure to comply was met with fines. It was noted that the collection of beetles on a massive scale reduced damage but could not seriously reduce the numbers of the beetles [28]. There was also the danger of the beetle “getting out of hand” during labour shortages. Hopkins went on to call for the “establishment of a natural check” for CRB [28]. Quarantine measures were introduced in American Samoa to prohibit the introduction of wood or organic materials that might contain the beetle [29]. With these somewhat drastic measures in place, Samoa was able to continue to produce sufficient coconuts for local consumption and sufficient copra for export. Simmonds reported that the very high levels of damage observed in the early years of the outbreak had been reduced by constant trapping, but damage could still be severe in some areas [30]. Very high levels of damage and coconut plant death in neglected plantations were reported by Cumber [31].

The beetle invasion was followed by disruptions of WWI, the change of Government administrations, and the tragedy of the 1918 influenza epidemic in Samoa. These events affected the CRB control programme, and the trapping and *Metarhizium* campaigns were abandoned [28]. Compulsory work for sanitation and the removal of breeding materials backed by beetle inspectors and fines for non-compliance remained the main strategy for CRB control in Samoa. The Rhinoceros Beetle Ordinance [9] strengthened the regulations and clarified the inspection system.

#### 1.1.3. Search for Effective BCAs

Biological control continued to be the long-term objective for control of CRB on the Samoan islands. In 1945, more than 400 parasitic wasps (*Scolia ruficornis*), imported from Zanzibar (East Africa), were released, and tested against CRB [30]. The wasp successfully established itself in the introduced region but failed to reduce the CRB population to a sub-economic level [12,13]. The viral pathogen, OrNV, isolated and identified by Huger in 1963, was first introduced and tested in Samoa in 1967 [15,32]. Virus-infected CRB larvae were imported from the laboratory of the Biologische Bundesanstalt in Darmstadt, Germany. For mass production, healthy CRB grubs collected in Samoa were fed with a mixture of sawdust and infected gut macerates. Around 1500 dead infected grubs were then applied to the breeding sites (e.g., rotten coconut logs) in different islands (e.g., Manono, Savai’i) within the country. The first field- collected larvae infected with the virus were confirmed from Upolu in Oct 1968, and within two years of release the virus successfully spread through CRB populations of Upolu and Savai’i, with a reported infection rate of 73% and a dramatic reduction in palm damage [32]. Quantitative studies before and after virus introduction reported adult infection rates of 35–40% after about two years [33,34].

During the mid-1970s, increases in CRB populations and palm damage were reported in several outbreak areas within Samoa [35]. This was largely due to a high number of breeding sites and lack of plantation sanitation, where the natural level of virus was considered insufficient to control those outbreaks. In response, the virus was re-released into outbreak areas during 1975–1979 [35]. Following the releases, adult CRB populations dropped from 30–50% at the start to 10–30%, whereas damage to central fronds was reduced from 70% to 30% at one site. Stechman and Semisi reported persistent infection in field-collected beetles, fluctuating from 27% to 45% from 1976–1983 [36]. The application of macerates-infected grubs into breeding sites was still the preferred application method. Later, it was found that the beetles themselves could be used as “flying virus factories”, and sites could be colonized by the virus by the release of as few as 50 beetles artificially infected with the virus into a CRB-infested area [15].

Research on *M. anisopliae* was revived during the 1960s by Marschall, who produced fungal spores on agar and applied them to heaps of breeding material and reported 60–100% infection [37]. Latch and Falloon continued this work and reported sites with high levels of infection, and even the “complete annihilation” of CRB from compost pits treated with the Samoan isolate and another long-spored isolate of *M. anisopliae* [38]. Although high levels of control could be obtained from direct application, there was little spread of the fungus from the sites of application, limiting the use of the fungus to accessible and concentrated breeding sites.

### 1.2. Status of CRB in Samoa after 100 Years

In Samoa, CRB has been managed through an integrated system relying on sanitation and BCAs, as described by UNDP-FAO [39]. This IPM approach has been maintained with support from the Samoan Government through the Ministry of Agriculture and Fisheries (MAF) for assistance with sanitation, damage assessment, and the application of BCAs where necessary. Damage to tall palms has been maintained at reasonable levels (<30% of the palms with first four fronds damaged) except in “hot spots” with substantial amounts of breeding material [40] (Supplementary Materials S1). A beetle survey in 2007 indicated that OrNV remains widespread in the CRB population, with 94.4% (51/54) of beetles collected positive for the virus after PCR testing [41] (Supplementary Materials S2), although this may be an overestimate from cross-contamination in traps. Further beetle collection in 2015 indicated 70.0% (21/30) OrNV positive by PCR (Supplementary Materials S2), and recent results are presented below. Anecdotal reports suggest that damage has not been excessive despite a high number of beetles in pheromone trap catches, probably reflecting the weakened state of beetles infected with the virus.

Most of the coconut palms in Samoa are old, with yields insufficient to meet the demand for new products from the coconut industry such as virgin oils, coconut water, and organic soaps [42]. In response to this increasing demand for coconut products and the needs for local food security, the Samoan Government has prioritized the renovation of the coconut industries and promoted the replanting of coconuts. More than 300,000 coconut and 300,000 cocoa seedlings have already been planted around the country, which will not only benefit the industry but has also helped laid-off workers in the Samoa tourism industry hit hard by COVID-19 lockdowns [43]. A coconut germplasm collection centre is also being developed to ensure access to diversity of the crop [44]. However, these plans are under serious threat from the growing population of CRB resulting from the felling of old palms and changes in cropping systems [45]. Furthermore, the coconut industries are also threatened by the rapid spread of an invasive CRB haplotype (CRB-G), which is devastating palms in surrounding PICTs [8,21,43]. To evaluate the current threat and utilize modern technologies in the evaluation, a new survey for CRB and incidence of OrNV on Upolu, Samoa was carried out.

## 2. Monitoring of CRB in Upolu and Determination of Haplotype and OrNV Incidence

### 2.1. CRB Monitoring

**Figure 4:**
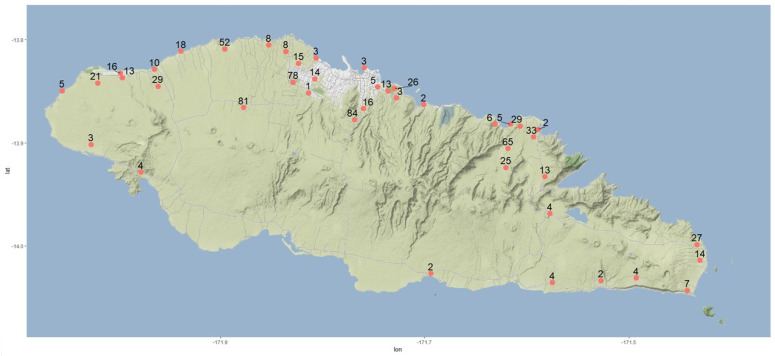
CRB (*Oryctes rhinoceros*) monitoring sites in Upolu, Samoa. Red dots indicate the trap location with trap catch numbers from individual traps indicated. Longitude values are shown on the x-axis and the latitude values on the y-axis.

A study was conducted in Upolu, Samoa during May–July 2020. A network of 42 pheromone bucket traps around the island were established to monitor CRB populations (Figure 4). The traps were kept in villages and plantations. Each trap was baited with the synthetic attractant oryctalure (ethyl 4-methyloctanoate; ChemTica Internacional, Costa Rica) and hung in the tree canopy or in a wooden pole at approximately 1.5 to 1.8 m above the ground. The traps were emptied, and beetles were removed approximately monthly. As there was considerable variability in the days each trap was operated, the total trap catch number was converted to ‘beetle catch per 30 days’ (i.e., month) to allow comparisons between sites. The numbers of beetles were recorded and sexed and analysed as CRB adults/trap/month [22].

Beetles were trapped throughout the trap network with considerable variation among traps (Figure 4). The number of adults/trap/month ranged from 1 to 84, with an average of 19.0 ± 0.52 adult/trap/month. Highest numbers were recorded from the traps established in the periphery of Apia (central north coast in the map with a white background). The surge in CRB population in this area is likely due to a greater availability of larval feeding resources (dead palm stems) from the felling of old palms because of the replantation program and urbanization. In 1972, a considerably lower number of beetles (an average of 5.15 ± 0.46 adult/trap/month) was recorded by trapping when natural CRB populations in Apia were low due to the early impact of the virus [46]. The experiment was, however, conducted using vane traps and two different chemical attractants (ethyl dihydrochrysanthemumate and ethyl chrysanthemumate).

In the current study, the south coast of the island had comparably lower catch numbers (average of 3.7 ± 0.59 adult/trap/month). Sixty-five percent of the beetles trapped were females, whereas 35% were males. This is consistent with previous studies where a higher rate of attraction of females toward the aggregation pheromone has been found [22,47,48]. Monitoring data from the pheromone traps suggest that there are several hotspots for CRB. Trap location was mapped using ‘R Studio’ software with data packages: “ggplot” and “ggmap” [49].

### 2.2. Determination of CRB Haplotype and Presence/Absence of OrNV

Following the methods used by Marshall et al., 100 CRB adult samples collected from the 42 pheromone traps were analysed to determine CRB haplotype (G or S) as well as the presence of OrNV [8]. One to three beetles were taken from each trap, placed in individual containers, and transported to the laboratory under the Ministry of Agriculture and Forestry (MAF, Apia, Samoa). Beetles were sexed and dissected, recording the condition of the gut. A 2 cm segment of the gut was removed and divided into two with the anterior segment placed in monopropylene glycol (MPG) for DNA analysis and the posterior section in formalin-aceto-alcohol (FAA) for histology. The gut samples were then sent to AgResearch, New Zealand for PCR analysis, sequencing, and histology. CRB haplotype (G or S) and presence or absence of OrNV were determined following the protocols described by Marshall et al. [8]. The haplotype clade was determined by sequencing a 676 base pair fragment of the mitochondrial cytochrome c oxidase subunit I (*COI*) gene and compared with the established database [8].

**Figure 5:**
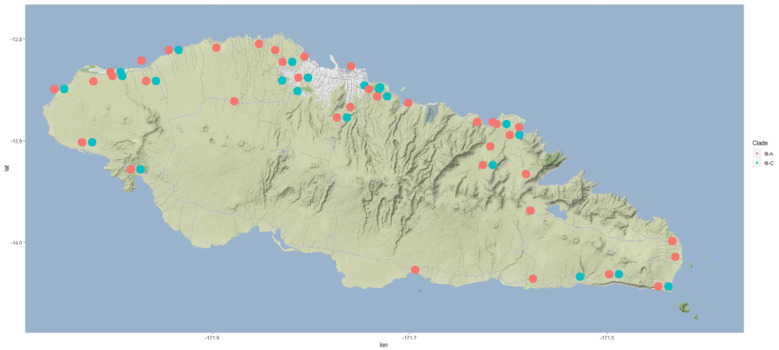
Distribution of assigned subclades from CRB (*Oryctes rhinoceros*) samples collected from the pheromone trap network in Upolu, Samoa. Color indicates different variants within clade III as per Marshall et al., 2017, with III-A (red dots) indicating specimens identical to the Samoa A15 reference sequence, while III-C (blue dots) specimens were identical to the Samoa A35 reference sequence. Longitude values are shown on the x-axis and the latitude values on the y-axis.

**Figure 6:**
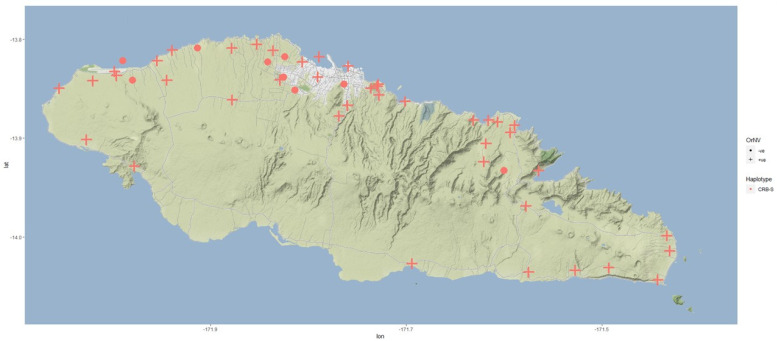
Determination of haplotype grouping as well as presence or absence of OrNV on CRB (*Oryctes rhinoceros*) samples collected from pheromone trap network in Upolu, Samoa. Red crosses indicate the traps from which the CRB samples were OrNV positive whereas red dots indicate traps with OrNV negative CRB samples. Longitude values are shown on the x-axis and the latitude values on the y-axis.

Based on the genetic analysis, all the 100 CRB specimens belong to CRB-S haplotype grouping using the PCR-RFLP analysis method (Table S1). This is consistent with the analysis of CRB samples from Samoa collected during 2015 [8]. None of the beetles were CRB-G haplotype, as detected within the outbreaks areas of Guam, Hawai’i, PNG, and Solomon Islands. Further, sequencing of the *COI* gene from 100 of these CRB-S specimens showed that all were identical to the two reference Samoan haplotypes described in clade III by Marshall et al. [8] (Figure 5). OrNV was confirmed from 91 of the 100 specimens, indicating the presence of virus. The remaining nine samples (two male, seven female) were negative for OrNV via PCR. The samples without virus were all from the north side of Upolu (Figure 6). Only one trap, with one beetle sampled, had no record of virus. The results indicate that the virus is widespread in the CRB-S population in Upolu, Samoa, as found previously [33,50]. The high level of virus infection rates (91%) is probably an overestimate, as the beetles were taken from traps containing other beetles where cross-contamination is a known problem [51]. The survey design can be modified to overcome this issue. Based on the visual diagnosis, 43% of the beetle gut indicated beetles infected with OrNV with characteristics symptoms of swollen gut. Eight of the nine beetle samples recorded as PCR negative showed no symptoms of disease during visual observation. Therefore, the visual diagnosis supports the PCR data, indicating that a high proportion of the CRB have the disease.

Of the 47 gut samples that were analysed for virus symptoms using histology, 44 of these samples matched OrNV PCR results (Table S1). Three samples were negative by PCR but showed virus symptoms by histology; these samples were positive for virus when the 1:10 and 1:100 dilution of extracted DNA was used. Histology was used as an independent method to confirm OrNV infection status, and the results showed 93% correlation with PCR.

## 3. Discussion and Future Recommendations

From its accidental introduction around 1909, CRB established, multiplied, and spread extremely quickly around the Samoan islands. In about 10 years, it had colonized all the palm growing areas of the country. CRB can be a strong flyer but generally does not disperse far if resources for feeding and breeding are available in the local area [52]. In Samoa, the beetle spread at a rate of about 16 km per year (80 km in 5 years), which is similar to the rates reported from other invaded Pacific islands [1,53]. This rapid rate of spread was potentially due to the healthy state of the beetles and high populations. It is possible that the spread, particularly between islands, was assisted by accidental human transport with planting materials, as beetle flights over long distances are unlikely. The rapid population increase and the rate of spread suggest that the founder population could have been made up of a high number of beetles, potentially with the importation of bulk plant materials and no quarantine services. Interestingly, DNA analysis indicated that the Samoan population of CRB consists of two closely related haplotypes separated by two nucleotide polymorphisms [8]. Given the slow evolution of the *COI* mitochondrial gene marker, both haplotypes could have been represented in the original invasive population [54]. There is no evidence of the subsequent introduction of additional haplotypes into Samoa, which indicates that the awareness of the CRB threat and the application of quarantine measures at the ports and other points of entry have been effective, to date, in keeping other variants out.

The isolated island environment of Samoa seems to have been highly favourable for the arrival of CRB. The early reports all remark on a high numbers of beetles in breeding materials and heavy damage to the palms [4,5,25]. In the initial stages of the infestation, “giant” specimens (57 mm: about 2.24 in length) were reported, but these were already rare in 1913, suggesting the impact of intraspecific competition as the population expanded [25]. Heavy damage, low biodiversity, and a lack of natural enemies led to the call for introduced BCAs. Despite the introduction of a high number of invertebrate BCAs to Samoa from other parts of the tropical world, few seem to have survived, and only the hymenopteran *S. ruficornis* are reported to have established themselves in any significant numbers [13,30].

Insect pathogens have been more successful as BCAs. Although *M. anisopliae* was isolated from CRB larvae native very early [25], it is likely that the fungus arrived with the invading CRB from Asia. The fungal spores illustrated by Friederichs are elongated (9–14 µm in length) and would now be defined as *M. majus*, a species often associated with dynastine scarabs [55,56,57]. Regardless of origin, the fungus has proven useful in Samoa, using a simple method of in vivo production and distribution of fungal-contaminated organic matter to fresh breeding sites [2]. Various researchers [25,38] have reported favourably on the methods and high levels of infection (up to 100%) after treating organic matter heaps with the fungus. The major challenge is to identify and treat breeding sites, which may be diverse in the mixed farming systems adopted by Samoan farmers. Fungal infections are frequently reported in field-collected CRB (M. Tupola, Personal communication). The natural spread of the fungus to untreated organic matter heaps after applying the Samoan strain of *M. anisopliae* has been reported from Tonga [38]. Since the discovery of *M. anisopliae* in the Samoan CRB population more than 100 years ago, several other *Metarhizium* species isolates have been introduced against CRB in Samoa. Further work is necessary to determine the most effective strains in the Samoan environment and then to incorporate these into the IPM programme.

The successful use of OrNV against CRB has been recognized as a landmark example of classical biological control [58]. The virus spread rapidly throughout the Samoan islands and persisted remarkably in the population [32,33,34]. The rapid spread of the virus appears to be aided by the aggregation behaviour of adults. However, visual diagnosis of the gut was used as the indicator of infection during those times and interpretation is dependent on the skill of the practitioner. Therefore, there was a need for a simple and reproducible technique to successfully integrate virus into pest management decision-making. The development of molecular biology during the 1990s allowed an objective PCR system to be developed for the detection of OrNV [59]. PCR analysis has confirmed the widespread presence of OrNV in the Samoan CRB population, although some of the positive PCR results may be from beetle-to-beetle contamination inside the trap rather than infection. In our recent study, PCR strongly correlated with visual diagnosis and histology but was much more likely to record infection. The OrNV has now been circulating among the CRB population for more than 50 years, and it is likely that some evolution may have taken place. Transmission may have been favoured to the cost of pathogenicity. DNA sequencing now allows us to analyse isolates and compare with the original strain and comparative regional isolates. The selection of more pathogenic strains may allow us to increase the efficacy of the biological control system.

Although *Metarhizium* and OrNV are the primary BCA options for CRB in Samoa, it is important that their use is not viewed in isolation. More than 100 years ago, Friederichs warned against considering the fungus as the unique solution for CRB control and stressed that it must be incorporated with sanitation, the destruction of breeding sites, and other control measures [25]. Similarly, after the international success of OrNV in spreading disease and reducing damage from CRB, Huger (2005) explained how the virus must be integrated into an IPM programme with other forms of management and control [15].

From the initial colonization of Samoa, CRB (clade III) has spread to other islands in the South-West Pacific (Tonga, Fiji, Wallace Island). In the period post-WWII, intensive research supported by the United Nations, FAO, and others led to a generally agreed IPM approach to CRB that could be implemented by the Pacific nations [39,60]. The first layer was to maintain awareness and quarantine systems to limit the spread of the pest to uninfested islands. The second was to understand the biology and limit the breeding cycle by cleaning up organic matter and dead palms, and the third level was to introduce and manage controls—virus, fungus, and chemicals, where necessary. Implementing the IPM system, coupled with the impact of the OrNV, has meant that CRB clade III has not expanded its range to more countries in the South-West Pacific in the last 50 years. Additionally, coconut production for food and as a cash crop has continued in the affected countries, and new economic opportunities have started to emerge.

In Samoa, new opportunities have arisen for coconut production with the increasing popularity of virgin coconut oils and coconut water. The Government of Samoa has prioritized the renovation of the coconut industries and promoted the replanting of coconuts to bolster trade and sustainable village economies [43]. However, the replantation program is under serious threat from the growing population of CRB in the replanting areas [61]. In several instances, the replantation program was delayed due to a very high incidence of the pest [61]. Most of the palms are around 2–3 years old, the phase where they will become most vulnerable to CRB. The increasing population of CRB is a substantial threat to the replantation program and could cause heavy palm losses if left uncontrolled. Therefore, there is a need to revisit the IPM programme to enhance the sustainable management of CRB.

After more than 100 years of CRB in Samoa, what are the lessons learnt, and how must we adapt for the future? The key lesson has been the need for integrated solutions (IPM), but how can these be adapted for the conditions of the 21st century? As always, the first response is awareness and quarantine. It is essential for Samoa to maintain a secure quarantine system to prevent the invasion of new variants of CRB such as the highly damaging CRB-G [8]. Access to molecular diagnostics must be maintained, as there are no clear morphological features to distinguish between CRB variants. The key to the management of any CRB variant will continue to be the elimination of heaps of organic matter with potential to form breeding sites for the beetle, although as Bedford (1980) noted, the methods are “laborious, expensive, unpopular and frequently ignored” [1].

Biological control by OrNV provides a level of control and appears to be weakening the beetle, limiting damage, and preventing further spread. As the virus has recycled and evolved in the Samoan islands for more than 100 years, it is timely to examine the genetics of the virus and determine whether the retesting and reintroduction of new isolates are warranted. A similar investigation should be carried out with *Metarhizium* spp. to ensure the use of the best isolates. When evaluating BCAs, it is vital that they are tested within an IPM context, in concert with other remediation procedures. Methods are now available to monitor and assess releases that should be used to ensure the ongoing success of the BCA release.

In coping with the invasion of CRB, the Samoan experience has contained some remarkable successes; the discovery and first use of a *Metarhizium* fungus against a tropical dynastine scarab pest; the development of a practical IPM system for CRB combining management and BCAs; and the first use of a self-perpetuating insect virus in regional control of CRB. From these successes, there are lessons to be learned for all on managing invasive pest threats, particularly those from resource-limited island communities, and will include exclusion, elimination, containment, biological control with self-replicating agents, and IPM, depending on the stage of the invasion.

## Data Availability

Data are contained within the article and supplementary materials.

## Supplementary Materials

The following supporting information can be downloaded at: https://www.mdpi.com/article/10.3390/insects13050487/s1, Supplementary Materials S1: Samoa *O. rhinoceros* beetle damage assessment workshop—A report. Supplementary Materials S2: Screening of *O. rhinoceros* beetle gut samples from Samoa for the presence of OrNV—A report. Table S1: Summary table of haplotype, virus detection and histology results (excel table as a separate file).
