# Supplementary material for: Coconut Rhinoceros Beetle in Samoa: Review of a Century-Old Invasion and Prospects for Control in a Changing Future
Source: Insects. 2022 May 23;13(5):487. doi: 10.3390/insects13050487 (PMC9145595; doi:10.3390/insects13050487)
Supplement: Supplementary file 1 [file insects-13-00487-s001.zip › Supplementary Materials S1.pdf]

Samoa rhinoceros beetle damage assessment workshop

Nu'u Research Stn, MAF Samoa

Jan 20-24, 2014

Workshop Report

Trevor Jackson

AgResearch, Lincoln

Contents

1. Outcomes and recommendations
2. Workshop introduction
3. Review of rhinoceros beetle in the Pacific and update on the problem of rhino beetle in the target countries.
4. Use of digital cameras and GPS
5. Development of a standardised method for damage assessment for use in the project

1. Outcomes and recommendations

- Distribution of rhinoceros beetle was reviewed. Concern was raised over highly damaging populations in Guam and Port Moresby and also the recently discovered incursion into Hawaii
- Palm damage assessment systems were reviewed. Four different systems were assessed in the field and a uniform system for damage assessment recommended.
- Digital cameras with GPS were provided to four groups, Fiji MAF, Samoa MAF, PNGOPRA and SPC. Basic instructions on use were provided and all registered users were considered competent by the end of the workshop.
- Instruction on mapping of survey data, including photos, was provided using Google Maps and Picasa systems. It is essential that team members have access to these programmes on their computers for work and that good internet connections are available.
- The process of beetle gut extraction for virus analysis was reviewed by the team. Use of photography using the digital cameras to aid diagnosis was tested.
- Plans were developed for damage assessment surveys in the three affected countries
- Links were made between team members and plans developed for collaboration.

Specific recommendations for Samoa

- A pilot damage survey should be initiated on Upolu covering 20 sites.
- Fresh virus strain X2B should be tested against Rhino beetle adults using standard methods.
- An experiment should be established at Nu'u to test impact of current methods for treatment of breeding heaps for beetles.
- A review meeting should precede implementation of a national survey of RB damage

General recommendations

- Similar pilot surveys should be initiated in Fiji and PNG
- Teams should collaborate through sharing methods for surveys, bioassays and IPM implementation.
- The programme team should coordinate for planning, analysis and publication of results

Workshop introduction

A specific workshop on damage assessment had been suggested during planning sessions in 2014 and was kindly hosted by MAF Samoa. It was planned to include representatives from SPC and the PICT groups working with the rhinoceros beetle biocontrol programme. The meeting was held at MAF Crops Division headquarters from Jan 20-24, 2014.

Workshop objectives from plan (9 Jan 2014)

- *To update the team on the situation of rhino beetle in the Pacific.*
- *To update team members on the problem of rhino beetle in each of the target countries (and research/control work being undertaken) .*
- *To review damage assessment methods in use and highlight strengths and weaknesses.*
- *To develop and validate a standardised method for damage assessment with the team members.*
- *To test use of digital cameras and GPS in damage mapping and confirm standardised methods for team members.*
- *To review data from pheromone trapping as a population monitoring tool.*

Trainers for workshop

**Name**	**Institute, email**
**Trevor Jackson**	**AgResearch, New Zealand (trevor.jackson@agresearch.co.nz)**
**MacLean Vaqolo**	**SPC LRD (macleanv@spc.int)**
**Aradhana Deesh**	**MPI Fiji (Aradhana.deesh@agriculture.govfj)**
**Solomon Sar**	**PNGOPRA (solomon.sar@pngopra.org.pg)**

RHINO BEETLE TRAINING WORKSHOP 2014

PARTICIPANTS LIST

No.	Name	Organization	Position	Email/ph#	Signatures
1	Trevor Jackson	Agr Research N.Z			
2	Maclean Vaqalo	SPC	Entomologist	macleanV.@spc.int	+679 939 3355
3	Aradhana	MAF Fiji	Technical Assistant Entomology Unit Plant Protection	aradhana.deoh@agriculture.gov.fj (+679) 9224819	[illegible]
4	Solomon	PNG			
5	Dr. Seuseu	MAF - Samoa	Consultant	7261250	
6	Fa'ava Siaunu'ua	MAF - Samoa	Research officer	7788249	[illegible]
7	Fuifatu Billy Enosa	MAF - Samoa	Senior Research (Entomology)	7611941	[illegible]
8	Juvita Tone	MAF - Samoa	Research officer	juvita.tone@maf.gov.ws. 7653774	[illegible]
9	Kuatemane Tuapola	MAF – Samoa	Research officer	7289030	[illegible]
10	Sailo Pao	MAF - Samoa	SCAO	7230442	
11	Tommy Tu'uamalii	MAF - Samoa	SCAO	7727692	[illegible]
12	Angelika Tugaga	MAF - Samoa	Senior Research (Pathology)	7245035	[illegible]
13	Aualiitia Parate	MAF - Samoa	PRO		
14	Tupito Moananu	MAF - Samoa	Information officer	7720836	
15	Taugata Seumanutafa	MAF - Samoa	Research officer	7227062	TS.
16	Fa'alelei Tunupopo	MAF - Samoa	Research officer		
17	Fata A Fania	MAF - Samoa			

email

Fuifatu Enosa – billy.enosa@maf.gov.ws

Workshop agenda

Date	Activities
**20 Jan**	**Welcome from MAF, Nu'u, Review of rhinoceros beetle status and activities**
**21 Jan**	**Visit to Nu'u labs. Activity planning. Introduction to digital cameras**
**22 Jan**	**Field visit for damage survey**
**23 Jan**	**Developing damage assessment methods and systems**
**24 Jan**	**Review of methods, workshop closure**

3. Review of rhinoceros beetle in the Pacific and update on the problem of rhino beetle in the target countries.

The distribution of rhinoceros beetle through the Pacific was discussed and current locations compared with earlier maps (Jackson ppt, See below). The outbreaks in Guam, Port Moresby, and now Hawaii, are causing concern, especially as the Guam population is proving difficult to infect with virus. An updated map of distribution including the beetle genotype and presence of virus is required and will be prepared under Section 3 of the revised programme

Presentations (Appendix 1)
Trevor Jackson. Rhinoceros beetle, status update in the Pacific. AgResearch/SPC workshop Samoa, 20-24 January 2014.
Aradhana Deesh. Rhinoceros beetle in Fiji
Solomon Sar. Managing *O. rhinoceros* in New Ireland

4. Use of digital cameras and GPS

The team were introduced to the use of digital cameras and GPS. Four PENTAX WG3 GPS cameras were issued to the participating teams

Camera number	Issued to	Person responsible
1	SPC - LRD	Maclean Vaqolo
2	MAF Samoa	Angelika Tugaga
3	MPI Fiji	Aradhana Deesh
4	PNGOPRA	Solomon Sar

A brief training was given and users agreed to share information on the best ways of using the camera and assist each other to overcome problems.
Instructions for use are provided in the accompanying Quick Guide or the Operating Manual booklet.

Two batteries were provided with each camera. These need to be charged fully overnight before going out into the field as using the GPS takes extra energy.
The GPS unit in the camera can be turned off when not recording locations. This can be done on Settings Screen 4.
For managing photos see Tutorial **Managing Pentax photos from the WG-III digital camera with GPS labels (Appendix 3)**

5. Development of a standardised method for damage assessment for use in the project

The need for methods of damage assessment were discussed and it was agreed that a standard method is required to be able to compare impact of rhinoceros beetle between countries and regions and also provide a method for establishing a damage baseline for evaluation of the benefits of IPM interventions.

The process of beetle feeding to produce damage was reviewed in the workshop (See Jackson Damage, Powerpoint) and methods that have been used for damage assessment discussed. Five techniques were defined.

- Leaf knotching as an indicator of RB feeding
- Percentage of palms damaged
- Damage to the top four fronds
- Percentage of damage to the top 4 fronds
- Upper and lower crown damage
- Number of fronds damaged
- Grading of damage to the crown on a 1-5 scale.

(For details see Appendix)

A team evaluation was prepared to assess five different assessment systems at five distinct locations. At each location, a predetermined site where palms could be observed from a roadway or path, a sample of 50 palms were assessed taking all the palms in the area for small plantation or every 2nd or 3rd palm in more numerous stands. The methods used were;

- Percentage of palms damaged
- Damage to the top four fronds
- Percentage of damage to the top 4 fronds
- Upper and lower crown damage
- Grading of damage to the crown on a 1-5 scale.
- Estimate of % damage (leaf loss) to the crown of the palm

In addition GPS tagged digital photography was used to map the sites and record level of damage.

Some output from the assessment is included below and a sample template included as Appendix 2.

Rhino beetle survey (Test output)

Site Mulifuanua

Date 22 January 2014

GPS Latitude	13 50\'3.1" S
GPS Longitude	172 1\'57.8" W
GPS Altitude	20.2

Site description

To be provided

Photos downloaded to;

MAF Samoa/RB Survey/Jan 2014/Site 4 Mulifanua

Saved screenshots from Picasa

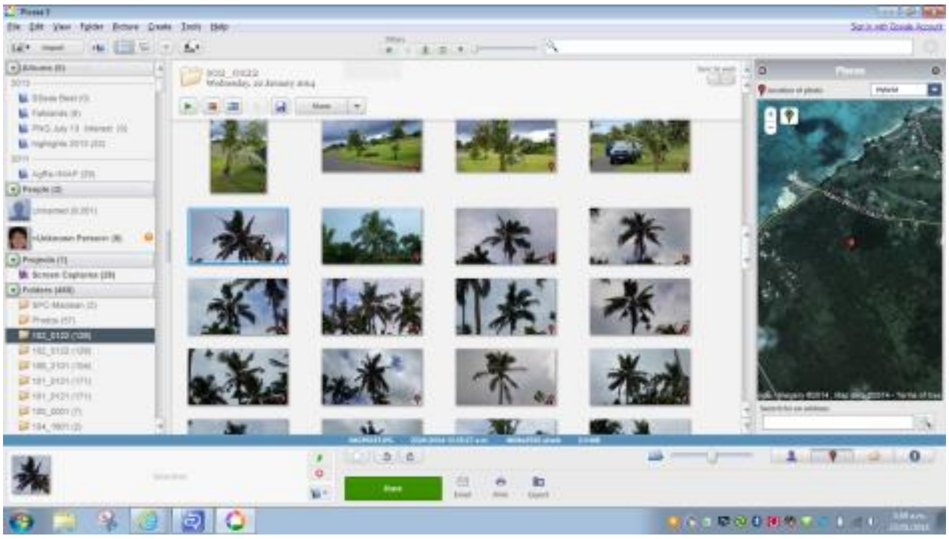

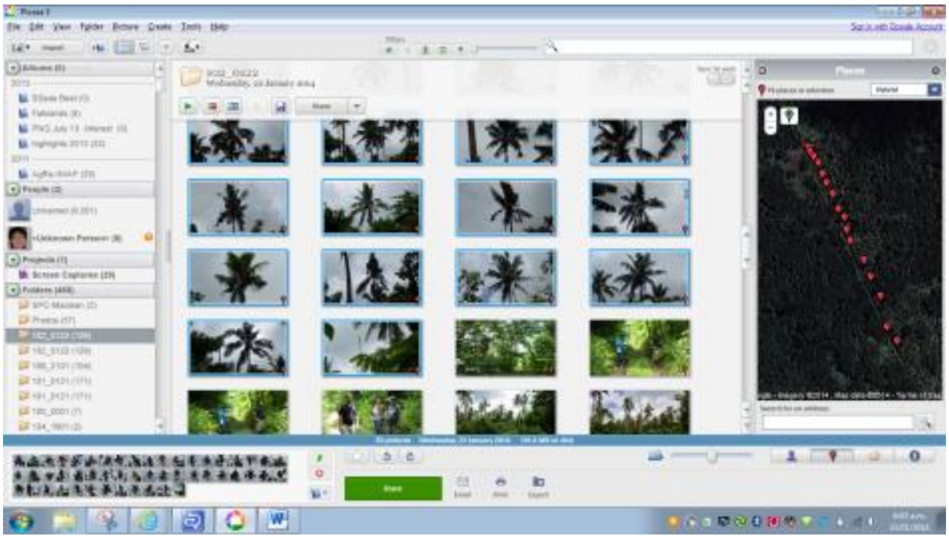

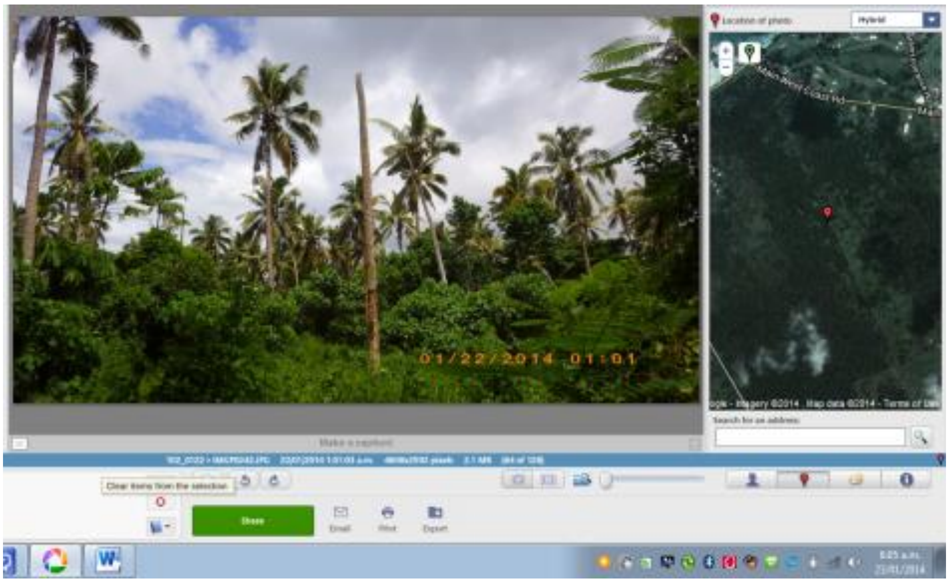

As shown above, by using the tagged photos the surveyed locations could be fixed and the route of the survey determined. The results from each group for % palms damaged and % of the top four fronds damaged were collated and shown in the table below.

Summary for Rapid Damage Survey Upolu Island January 2014

Location	Date	No. of palms counted	Team	No. of palms damaged	No. of palms had first four fronds damaged	% Damage of palms	% Damage for first four fronds
**Mulifanua**	22/01/2014	50	AD	49	38	98	76
			Mane	43	41	86	82
			SSar	50	46	100	92
**Nofoalii**	22/01/2014	50	AD	48	38	96	76

			Mane	40	40	80	80
			SSar	46	41	92	82
**Sa'anapu/Safata**	22/01/2014	50	AD	35	23	70	46
			Mane	40	34	80	68
			SSar	41	35	82	70
**Leulumoega-tuai**	22/01/2014	50	AD	38	23	76	46
			Mane	37	26	74	52
			SSar	46	27	92	54
**Aleisa 1**	22/01/2014	50	AD	33	19	66	38
			Mane	25	20	50	40
			SSar	42	25	84	50

The results were discussed. Both methods appeared to be reasonably consistent at sites with high damage but were more variable for the whole palm assessment at the lower damage sites. The data will be further analysed.

The team suggested further comparisons to standardise observations and taking a digital photograph of each of the palms in each assessment.

An improved system was then tested using the coconut plantation at the Nu'u research centre. Four teams each carried out a damage assessment and produced a report on the block. A sample report is included.

Appendices

Appendix 1. Presentations AgResearch/SPC workshop Samoa, 20-24 January 2014.

Trevor Jackson.   Rhinoceros beetle, status update in the Pacific.
Aradhana Deesh.   Rhinoceros beetle in Fiji
Solomon Sar.   Managing *O. rhinoceros* in New Ireland
Trevor Jackson. Rhinoceros beetle damage assessment.

Appendix 2. Sample template for damage assessment

Appendix 3. Managing Pentax photos from the WG-III digital camera with GPS labels

Appendix 6. Pacific Biocontrol 2014

Appendix 6. Pacific Biocontrol 2014
