# Supplementary material for: Coconut Rhinoceros Beetle in Samoa: Review of a Century-Old Invasion and Prospects for Control in a Changing Future
Source: Insects. 2022 May 23;13(5):487. doi: 10.3390/insects13050487 (PMC9145595; doi:10.3390/insects13050487)
Supplement: Supplementary file 1 [file insects-13-00487-s001.zip › Supplementary Materials S2.pdf]

Screening of *Oryctes* Beetle gut samples from Samoa for the presence of *Oryctes* Virus

Jana Lottmann, AgResearch Lincoln

6 July 2007

Oryctes beetle gut samples (n = 250) were sent for screening for the *Oryctes* virus in February, March and June 2007.

Method

For the gut samples stored in ethanol, approx. 3mm pieces were ground using a sterile micropestle in 180µl of ATL buffer (Qiagen DNeasy Tissue Kit, Biolab Scientific QIA69504). The method was continued as per the kit instructions. At the final step the sample was eluted in 100µl of the elution buffer and stored at 4°C or -20°C long term.

PCR was set up using two different primer pairs specific to the *Oryctes* virus (OrV3a / OrV15b, and OrV15a /OrV15b). Positive and negative controls were included in the PCR set-up to ensure confidence in the results. The presence of *Oryctes* DNA resulted in a 945 bp fragment. BSA was included in the PCR reaction mix to reduce the inhibitory affects caused by the presence of humic acids or other inhibitors.

Results

In total, 54 gut samples were screened for the presence of *Oryctes* virus. According to the description of the samples (sex, and the condition of the insect and its gut), samples from all categories were screened for the presence of the virus. There was no sample sheet for the second lot (no. D1 to D50) but according to the appearance different samples have been analysed.

So far, only three samples (A6, E11, and E25) were negative in the PCR. Except for six samples (A19, A26, B14, C31, C44, and E44) which gave a weak PCR product, all other samples gave a strongly positive result in the PCR. In all cases the PCR negative control was negative.

The results showed that either primer pair could be used to detect OrV. There was no difference in the intensity of bands.

The PCR result are shown in the following tables:

Insect	Site No.	Trap No.	Collection Date	Sex	Dissection Date	Insect and gut condition	PCR Results
A1	Vaitele		01-Nov-06	F	02/Nov/2006	Female, live, Gut thin and dark with attached fat	
A2	Vaitele		30-Oct-06	F	02/Nov/2006	Mature female dead with some tissue deterioration, eggs in oviduct. Gut dark and thin.	
A3	Vaitele		30-Oct-06	F	02/Nov/2006	Female dead slightly swollen milky.	
A4	Vaitele		03-Nov-06	M	03/Nov/2006	Live male, Gut milky at front & dark rear	
A5	Vaitele		03-Nov-06	F	03/Nov/2006	Female, live, gut dark, lots of eggs.	
A6	Vaitele		06-11-06	F	10/Nov/2006	Milky gut, dead, egg laden	Ø
A7	Vaitele		06-11-06	F	10/Nov/2006	Dead, slightly swollen, milky, lots of eggs.	
A8	Vaitele		06-11-06	F	16/Nov/2006	Young female, dead, gut dark thin with fat attached.	
A9	Vaitele		10-11-06	F	16/Nov/2006	Dead, gut dark thin full of organic matter.	
A10	Vaitele		06-11-06	M	16/Nov/2006	Dead, gut swollen, milky.	
A11	Vaitele		10-11-06	M	10/Nov/2006	Mature beetle, dead, gut swollen and milky.	
A12	Vaitele		10-11-06	F	16/Nov/2006	Dead, gut slightly swollen, milky.	
A13	Vaitele		10-11-06	M	16/Nov/2006	Mature, dead, gut swollen milky.	
A14	Vaitele		10-11-06	M	16/Nov/2006	Mature, dead, gut thin, dark, full of organic matter.	+
A15	Vaitele		10-11-06	F	16/Nov/2006	Dead, gut slightly swollen, clear.	+
A16	Vaitele		10-11-06	F	16/Nov/2006	Dead, gut thin, dark, few eggs in oviduct.	
A17	Vaitele		10-11-06	F	16/Nov/2006	Dead, gut thin, dark, full of organic matter.	+
A18	Vaitele		10-11-06	F	16/Nov/2006	Dead, gut thin, dark with fats attached.	
A19	Vaitele		13-11-06	F	17/Nov/2006	Live, gut thin, dark, few eggs in oviduct	(+)
A20	Vaitele		13-11-06	F	17/Nov/06	Live, mature, swollen white and empty, egg laden.	+
A21	Vaitele		13-11-06	F	17Nov/06	Dead, mature, gut thin, dark and full of organic matter.	
A22	Vaitele		13-11-06	M	17/Nov/06	Live, mature, gut slightly swollen, milky.	
A23	Vaitele		13-11-06	F	17/Nov/06	Live, female, gut slightly swollen, milky, egg laden	
A24	Vaitele		13-11-06	M	17/Nov/06	Live, gut thin, dark full of organic matter	
A25	Vaitele		13-11-06	F	17/Nov/06	Live, mature, slightly swollen, milky.	

Insect	Site No	Trap No	Collection Date	Sex	Dissection Date	Insect and gut condition	PCR Results
A26	Vaitele		22-11-06	F	23/Nov/06	Mature, gut thin, dark, egg few egg in oviduct.	(+)
A27	Vaitele		22-11-06	M	23/Nov/06	Live, swollen white and empty	+
A28	Vaitele		22-11-06	M	23/Nov/06	Live, gut thin, dark.	
A29	Vaitele		22-11-06	F	23/Nov/06	Live, mature, no egg, gut slightly swollen, milky.	
A30	Vaitele		22-11-06	M	23/Nov/06	Dead, gut swollen, milky.	+
A31	Vaitele		22-11-06	F	23/Nov/06	Young gut swollen white and empty	
A32	Vaitele		22-11-06	F	23/Nov/06	Young, gut, swollen white and empty, no eggs.	
A33	Vaitele		22-11-06	F	23/Nov/06	Live, gut thin, dark and no eggs.	
A34	Vaitele		22-11-06	F	23/Nov/06	Live, mature, gut thin, dark no egg.	
A35	Vaitele		22-11-06	M	23/Nov/06	Live, mature, gut thin, dark	
A36	Vaitele		22-11-06	F	23/Nov/06	Live, young, gut, slightly swollen, milky, no egg.	+
A37	Vaitele		22-11-06	M	23/Nov/06	Live, mature, gut thin, dark	+
A38	Vaitele		22-11-06	F	23/Nov/06	Mature, gut slightly swollen, milky, no eggs	
A39	Vaitele		22-11-06	F	23/Nov/06	Live, mature, gut thin, dark, no egg	
A40	Vaitele		22-11-06	F	23/Nov/06	Live, gut thin, rear dark.	
A41	Vaitele		24-01-07	F	31/Jan/07	Live, mature, gut thin, clear, egg laden	+
A42	Vaitele		29-01–7	F	31/Jan/07	Dead, gut thin, dark full of organic matter.	
A43	Vaitele		29-01-07	M	31/Jan/07	Dead, young, gut thin, dark full of organic matter.	+
A44	Vaitele		29-01-07	F	31/Jan/07	Mature, dead, gut thin, dark few eggs in oviduct.	
A45	Vaitele		29-01-07	F	31/Jan/07	Young, dead, gut thin, dark no egg	+
A46	Vaitele		29-01-07	F	31/Jan07	Mature, live, gut thin, dark, no egg	
A47	Vaitele		29-01-07	M	31/01/07	Mature, live, gut thin, dark.	
A48	Vaitele		29-01-07	F	31/Jan/07	Mature, dead, gut thin, dark full of organic.	
A49	Vaitele		29-01-07	F	31/Jan/07	Dead, gut thin, dark and full of organic matter.	
A50	Vaitele		31-01-07	F	31/01/07	Mature, live, gut thin clear.	+

Insect	Site No.	Trap No.	Collection Date	Sex	Dissection Date	Insect and gut condition	PC Re
B1	Mulifanua	4	13/11/06	F	21/11/06	Live, Gut dark, slightly swollen with few eggs.	
B2	"	4	"	F	"	Live, dark thin gut, lots of fats attached around and eggs in oviducts,	
B3	"	2	22/01/07	F	26/01/07	Pure, gut few eggs healthy.	+
B4	"	"	"	F	"	Live, normal gut.	
B5	"	3	"	F	"	Live, matured ,slightly swollen gut.	
B6	"	4	"	F	"	Live , dark thin and brown gut ,with fat attached.	
B7	"	4	"	F	"	Live, normal gut, some fat attached.	
B8	"	"	"	M	"	Live, healthy full organic matter.	
B9	"	"	"	F	"	Live, slightly swollen and brown gut few eggs.	
B10	"	2	29/01/07	F	31/01/07	Dead, slightly swollen gut, few eggs.	
B11	"	4	"	F	"	Dead, matured , dark thin gut , normal.	+
B12	"	"	"	F	"	Dead, dark thin gut with few eggs.	
B13	"	3	12/02/07	M	14/02/07	Live, matured dark thin gut.	
B14	"	"	"	F	"	Dead, thin and dark gut, healthy.	(+)
B15	"	"	"	F	"	Dead, matured slightly swollen, and brown gut.	
B16	"	"	"	F	"	Dead, young, healthy, dark thin gut.	
B17	"	"	"	F	"	Dead ,young, healthy, dark thin gut.	
B18	"	"	"	F	"	Dead, young slightly swollen and white gut.	
B19	"	2	"	M	"	Dead matured swollen white empty gut.	
B20	"	2	1022007	F	"	Mature, thin, dark healthy gut	

Insect	Site No.	Trap No.	Collection Date	Sex	Dissection Date	Insect and gut condition	PCR Resul
B21	"	"	"	F	"	Live, Gut thin and dark	
B22	"	"	"	F	"	Matured, slightly swollen brown gut, with eggs.	
B23	"	"	"	F	"	Live, milky swollen few eggs.	
B24	"	"	"	M	"	Live, dark thin full of organic matter.	
B25	"	"	"	F	"	Young, dark thin gut.	+
B26	Mulifanua	5	26/02/07	F	27/02/07	Live, dark thin gut, full of organic matter.	
B27	"	"	"	F	"	Live, matured swollen milky with eggs.	
B28	"	2	"	F	"	Live, thin gut.	
B29	"	5	"	F	"	Live, swollen white gut.	
B30	"	"	"	F	"	Live, matured organic matter and brown gut.	
B31	"	3	"	F	"	Live, swollen milky gut, with eggs.	+
B32	"	5	"	F	"	Live, matured slightly swollen white gut.	
B33	"	"	"	F	"	Live, matured , thin gut, dark with eggs.	
B34	"	5	06/03/07	F	07/03/07	Dead, brown thin gut, full of organic matter few eggs.	
B35	"	"	"	F	"	Dead, matured thin dark gut, full of organic matter.	
B36	"	"	"	F	"	Live, young brown gut, few eggs full of organic matter.	
B37	"	2	26/03/07	F	28/03/07	Live, dark gut few eggs.	
B38	"	6	23/03/07	F	"	Dead, slightly swollen gut.	+
B39	"	2	"	F	"	Live, dark gut few eggs organic matter.	
B40	"	1	"	F	"	Dead, slightly swollen gut.	
B41	"	4	02/03/07	F	03/04/07	Live, thin gut organic matter.	
B44							+
B49							+

Insect	Site No.	Trap No.	Collection Date	Sex	Dissection Date	Insect and gut condition	PCR Resu
C 1	Safata	4	23-01-2007	M	26/01/2007	Live, Gut thin and dark.	
C2	Safata	5	30-01-2007	M	31/01/2007	Dead, Gut dark and thin. Normal healthy gut.	
C3	Safata	5	30-01-2007	F	31/01/2007	Dead, Gut dark and thin. Healthy gut	+
C4	Safata	5	30-01-2007	F	31/01/2007	Young, dead. Gut dark thin	
C5	Safata	5	30-01-2007	F	31/01/2007	Dead, normal dark thin gut.	
C6	Safata	5	30-01-2007	M	31/01/2007	Dead. Gut dark and thin	
C7	Safata	5	30-01-2007	F	31/01/2007	Live. Gut slightly swollen, milky.	
C8	Safata	4	12-02-2007	F	14/02/2007	Live. Gut slightly swollen. White and empty. No eggs	
C9	Safata	4	12-02-2007	F	14/02/2007	Dead. Gut swollen. Milky white. No egg	
C10	Safata	1	12-02-2007	F	14/02/2007	Live. Gut slightly swollen, milky, egg laden.	
C11	Safata	5	12/02/2007	F	14/02/2007	Dead. Gut thin and dark. Egg laden	+
C12	Safata	4	26-02-2007	F	27/02/2007	Dead young. Gut dark and thin.	
C13	Safata	4	26-02-2007	F	27/02/2007	Live. Gut slightly swollen	
C14	Safata	4	26-02-207	F	27/02/2007	Live. Gut swollen, milky.	+
C15	Safata	4	26-02-2007	F	27/02/2007	Young female. Gut thin, dark. Full of organic matter.	
C16	Safata	4	26-02-2007	F	27/02/2007	Young live female. Gut dark, thin. Full of organic matter.	
C17	Safata	4	26-02-2007	M	27/02/2007	Dead. Gut swollen and milky	
C18	Safata	4	26-02-2007	M	27/02/2007	Mature live male. Gut slightly swollen and milky	
C19	Safata	4	26-02-2007	F	27/02/2007	Live female. Gut slightly swollen. And milky	
C20	Safata	4	26-02-2007	F	27/02/2007	Live mature. Gut slightly swollen. Milky.	
C21	Safata	4	26-02-2007	F	27/02/2007	Live young female. Gut thin and dark.	

Insect	Site No.	Trap No.	Collection Date	Sex	Dissection Date	Insect and gut condition	PCR Result
C22	Safata	4	26-02-2007	F	27/02/2007	Young live female. Gut dark and thin. Full of organic matter.	
C23	Safata	4	26-02-2007	F	27/02/2007	Live and slightly swollen gut.	
C24	Safata	4	26-02-2007	F	27/02/2007	Young live. Gut dark thin. Full of organic matter	
C25	Safata	4	26-02-2007	F	27/02/2007	Live slightly swollen, dark rear gut.	+
C26	Safata	2	26-02-2007	F	27/02/2007	Live female. Gut dark and thin with fats attached.	
C27	Safata	2	26-02-2007	F	27/02/2007	Live young female. Gut swollen and milky.	
C28	Safata	2	26-02-2007	F	27/02/2007	Live female. Gut thin,dark. Full of organic mater	
C29	Safata	2	26-02-2007	F	27/02/2007	Young live female. Gut slightly swollen, milky	
C29	Safata	2	26-02-2007	F	27/02/2007	Live female. Gut slightly swollen and milky, few organic matter.	
C30	Safata	2	26-02-2007	F	27/02/2007	Live female. Gut thin and dark.	
C31	Safata	1	26-02-207	M	27/02/2007	Live mature male. Gut thin and dark.	(+)
C32	Safata	1	26-02-2007	F	27/02/2007	Dead female. Gut white and empty.	
C33	Safata	1	26-02-2007	F	27/02/2007	Dead mature female. Gut dark rear. Few organic matter.	
C34	Safata	1	26-02-2007	F	27/02/2007	Dead female. Gut thin and daek.	
C35	Safata	2	26-02-2007	F	27/02/2007	Dead female. Gut white and empty	
C36	Safata	4	06-03-2007	F	07/03/2007	Dead young female. Gut thin and dark.	
C37	Safata	4	06-03-2007	F	07/03/2007	Dead young female. Gut thin and dark. Full of organic matter.	
C38	Safata	4	06-03-2007	F	07/03/2007	Dead young female. Gut thin and dark.	+
C 39	Safata	4	06-03-2007	F	07/03/2007	Dead female. Gut dark rear. Few organic matter.	
C40	Safata	3	06-03-2007	F	07/03/2007	Mature dead female. Gut thin and dark.	
C41	Safata	5	06-03-2007	M	07/03/2007	Mature dead male. Gut thin and dark with fats attach.	
C42	Safata	2	06-03-2007	F	07/03/2007	Dead female Gut thin and dark	
C43	Safata	1	26-03-2007	F	28/03/2007	Dead female. Gut dark and thin.	
C44	Safata	1	26-03-2007	M	28/03/2007	Live female. Gut dark and thin.	(+)
C45	Safata	2	02-04-2007	F	03/04/2007	Dead female. Gut slightly swollen. Milky.	
C46	Safata	4	02-04-2007	F	03/04/2007	Live mature female. Gut thin and dark. Full of organic matter.	
C47	Safata	2	02-04-2007	M	03/04/2007	Live male. Gut dark and thin.	
C48	Safata	4	11-04-2007	F	12/04/2007	Live female. Gut swollen and milky.	
C49	Safata	2	11-04-2007	F	12/04/2007	Dead female. Gut thin and dark. Full of organic matter.	+
C50	Safata	2	11-04-2007	F	12/04/2007	Dead female. Gut thin and dark. Egg laden.	

Insect	Site No	Trap No	Collection Date	Sex	Dissection Date	Insect and gut condition	PCR Results
D1	Falealili	1	15-11-2006	F	21/11/2006	Dead female, gut thin, dark full of organic matter.	+
D2	Falealili	3	15-11-2006	F	21/11/2006	Live young female, gut thin dark, few organic matter	
D3	Falealili	1	15-11-2006	M	21/11/2006	Live, gut swollen, milky.	
D4	Falealili	1	15-11-2006	F	21/11/2006	Live young female, gut thin, dark, few in oviduct.	+
D5	Falealili	3	15-11-2006	M	21/11/2006	Live mature male, gut slightly swollen, milky	
D6	Falealili	1	15-11-2006	F	21/11/2006	Live, mature, swollen and milky.	
D7	Falealili	1	15-11-2006	M	21/11/2006	Dead mature male, gut thin, dark.	+
D8	Falealili	1	22-11-2006	F	23/11/2006	Live female, mature gut thin, dark, normal.	+
D9	Falealili	5	22-11-2006	F	23/11/2006	Live, gut slightly swollen, brown few organic matter, few eggs in the oviduct, fat attached	
D10	Falealili	5	22-11-2006	F	23/11/2006	Live mature female, gut dark and thin, eggs laden.	
D11	Falealili	5	22-11-2006	F	23/11/2006	Live young female, gut slightly swollen, eggs laden.	+
D12	Falealili	5	22-11-2006	F	23/11/2006	Live young female, brown thin gut, few organic, egg in the oviduct.	+
D13	Falealili	5	22-11-2006	F	23/11/2006	Live female, eggs laden, gut dark thin.	+
D14	Falealili	3	22-11-2006	F	23/11/2006	Live mature female, gut dark, thin.	
D15	Falealili	3	22-11-2006	M	23/11/2006	Live male, gut slightly swollen, milky.	
D16	Falealili	3	22-11-2006	F	23/11/2006	Live female gut brown, slightly swollen.	+
D17	Falealili	3	05-02-2007	F	07/02/2007	Dead mature female, gut thin, clear no egg.	
D18	Falealili	3	05-02-2007	M	07/02/2007	Dead mature female, slightly swollen milky.	
D19	Falealili	3	05-02-2007	F	07/02/2007	Dead mature female, gut swollen, milky, egs laden	
D20	Falealili	3	05-02-2007	F	07/02/2007	Young female, gut dark, thin. Lots of organic matter.	
D21	Falealili	3	05-02-2007	F	07/02/2007	Gut dark, thin, lots of organic matter, no eggs.	+
D22	Falealili	3	05-02-2007	M	07/02/2007	Live mature, gut slightly swollen, brown with some fats attached.	

Insect	Site	Trap	Collection	Sex	Dissection	Insect and gut condition	PCR
	No	No	Date		Date	Results	
D23	Falealili	3	05-02-2007	F	07/02/2007	Dead matured female, gut dark thin, full of organic matter.	
D24	Falealili	3	05-02-2007	F	07/02/2007	Live female, gut thin, clear.	
D25	Falealili	3	05-02-2007	M	07/02/2007	Dead mature male Gut swollen white and empty	
D26	Falealili	3	19-02-2007	F	21/02/2007	Matured female, gut swollen, milky, Egg laden	
D27	Falealili	3	19-02-2007	F	21/02/2007	Live mature female. Gut thin, dark. Full of organic matter. No eggs	
D28	Falealili	3	19-02-2007	M	21/02/2007	Dead male. Gut swollen white and empty	
D29	Falealili	3	19-02-2007	F	21/02/2007	Dead mature female. Gut thin, dark full of organic matter	
D30	Falealili	3	19-02-2007	F	21/02/2007	Dead female. Gut slightly swollen. Egg laden	
D31	Falealili	3	19-02-2007	M	21/02/2007	Dead male. Gut thin, dark full of organic matter.	
D32	Falealili	3	19-02-2007	F	21/02/2007	Dead mature female. Gut swollen white and empty	+
D33	Falealili	4	19-02-2007	F	21/02/2007	Dead mature. Gut slightly swollen, brown. Egg laden	
D34	Falealili	4	19-02-2007	F	21/0220/07	Dead female. Gut swollen white and empty.	+
D35	Falealili	4	19-02-2007	F	21/02/2007	Female dead. Gut thin, dark. Full of organic matter.	
D36	Falealili	4	19-02-2007	F	21/02/2007	Live female. Gut slightly swollen, dark rear.	
D37	Falealili	4	19-02-2007	F	21/02/2007	Live female. Gut thin, dark. Egg laden.	
D38	Falealili	4	19-02-2007	F	21/02/2007	Dead mature female. Gut dark, thin, full of organic matter	+
D39	Falealili	4	19-02-2007	F	21/02/2007	Live female. Gut slightly swollen, milky. No egg	
D40	Falealili	4	19-02-2007	F	21/02/2007	Dead female. Gut slightly swollen, brown with few organic matter.	+
D41	Falealili	5	19-02-2007	F	21/02/2007	Dead female. Gut slightly swollen, milky. No egg	
D42	Falealili	5	19-02-2007	F	21/02/2007	Dead female. Gut dark, thin. No egg.	
D43	Falealili	5	19-02-2007	F	21/02/2007	Live female. Gut dark, thin. Egg laden	
D44	Falealili	1	06-03-2007	F	07/03/2007	Live female. Gut dark, thin. No egg	
D45	Falealili	1	06-02-2007	M	07/03/2007	Live mature male. Gut dark, thin full of organic matter	

Insect	Site	Trap	Collection	Sex	Dissection	Insect and Gut Condition	PCR
	No	No	Date		Date		Result
D46	Falealili	1	06-03-2007	F	07/03/2007	Live female. Gut swollen white and empty. No egg	
D47	Falealili	1	06-03-2007	F	07/03/2007	Dead female, Gut slightly swollen milky. Few eggs in the oviduct.	+
D48	Falealili	1	06-03-2007	F	07/03/2007	Dead female. Gut dark, thin. No egg	
D49	Falealili	1	06-03-2007	F	07/03/2007	Dead female. Gut dark, thin. Full of organic matter. No egg.	+
D50	Falealili	1	06-03-2007	F	07/03/2007	Dead female. Slightly swollen milky. Egg laden	

Insect	Site No.	Trap No.	Collection Date	Sex	Dissection Date	Insect and Gut Condition	PCR Results
E1	Tiavea	4	15-11-2006	F	21/11/2006	Dead mature female. Gut dark thin, full of organic matter. No eggs.	
E2	Tiavea	4	15-11-2006	M	21/11/2006	Dead mature male. Gut dark thin.	
E3	Tiavea	1	15-11-2006	F	21/11/2006	Live young female. Gut dark rear. Slightly swollen. No eggs	+
E4	Tiavea	1	15-11-2006	M	21/11/2006	Dead mature male. Gut thin, clear.	
E5	Tiavea	1	15-11-2006	F	21/11/2006	Live female. Gut thin, clear. Eggs deposit in the oviduct.	
E6	Tiavea	1	15-11-2006	F	21/11/2006	Gut thin, dark. Lots of fat attached. No eggs.	
E7	Tiavea	1	15-11-2006	F	21/11/2006	Live female. Gut slightly swollen, milky. No eggs.	
E8	Tiavea	1	15-11-2006	F	21/11/2006	Mature female. Gut slightly swollen, milky. Egg laden.	
E9	Tiavea	1	15-11-2006	F	21/11/2006	Young live female. Gut swollen, white.	
E10	Tiavea	1	15-11-2006	F	21/11/2006	Young female. Gut slightly swollen, milky. No eggs	
E11	Tiavea	5	22-11-2006	F	23/11/2006	Live female. Gut thin, dark. Few eggs in the oviduct.	neg.
E12	Tiavea	5	25-01-2007	M	26/01/2007	Dead female. Gut thin, dark.	
E13	Tiavea	5	25-01-2007	M	26/01/2007	Dead male. Gut thin, clear.	
E14	Tiavea	5	25-01-2007	M	26/01/2007	Live mature male. Gut thin, dark.	+
E15	Tiavea	3	25-01-2007	F	26/01/2007	Dead young female. Gut thin clear. No eggs	
E16	Tiavea	4	05-02-2007	M	07/02/2007	Mature male. Gut thin, dark.	
E17	Tiavea	4	19-02-2007	F	21/02/2007	Dead female. Gut slightly swollen, milky.	
E18	Tiavea	4	19-02-2007	F	21/02/2007	Live female, Gut swollen, milky. Egg laden.	
E19	Tiavea	4	19-02-2007	F	21/02/2007	Live young female. Gut thin, dark.	
E20	Tiavea	4	19-02-2007	F	21/02/2007	Dead mature female. Gut thin, dark, full of organic matter.	
E21	Tiavea	4	19-02-2007	F	21/02/2007	Dead young female. Gut swollen white and empty. Eggs in the oviduct.	
E22	Tiavea	4	19-02-2007	F	21/02/2007	Mature live female . Gut thin, dark full of organic matter.	
E23	Tiavea	4	19-02-2007	F	21/02/2007	Live mature female. Gut thin dark	
E24	Tiavea	4	19-02-2007	M	21/02/2007	Live mature male. Gut slightly swollen. Dark rear.	
E25	Tiavea	2	19-02-2007	F	21/02/2007	Dead mature female. Gut thin, dark. Full of organic matter.	neg.

Insect	Site No.	Trap No.	Collection Date	Sex	Dissection Date	Insect and Gut Condition	PCR Result
E26	Tiavea	2	19-02-2007	F	21/02/2007	Dead mature. Gut thin, dark.	
E27	"	2	19-02-2007	F	21/02/2007	Dead young . Gut thin, dark.	
E28	"	2	19-02-2007	M	21/02/2007	Dead. Gut slightly swollen. Milky	
E29	"	2	19-02-2007	M	21/02/2007	Live. Gut swollen and white.	
E30	"	2	19-02-2007	F	21/02/2007	Dead mature. Gut thin, dark. Full of organic matter.	
E31	"	1	19-02-2007	F	21/02/2007	Dead, Gut slightly swollen.	+
E32	"	1	"	M	"	Dead, matured & slightly swollen milky gut.	
E33	"	1	05/03/07	M	07/03/07	Dead, thin gut & full of organic matter.	
E34	"	1	"	F	"	Dead, milky, swollen, white gut & few eggs.	
E35	"	1	"	M	"	Live, thin, brown gut.	
E36	"	1	"	F	"	Dead, thin, brown gut.	
E37	"	1	"	F	"	Dead, thin , brown gut & few eggs.	
E38	"	1	"	F	"	Dead, matured, thin, dark gut with eggs.	+
E39	"	1	"	F	"	Dead ,dark gut full of organic matter & few eggs.	
E40	"	1	"	F	"	Live, milky white gut with eggs.	
E41	"	1	"	F	"	Dead, matured, full of organic matter and brown gut.	
E42	"	1	"	F	"	Live, young, dark, thin gut with organic matter and eggs.	
E43	"	3	"	M	"	Dead, white, empty swollen.	
E44	"	2	"	F	"	Dead, dark thin full of organic matter.	(+)
E45	"	2	"	F	"	Dead, dark , few eggs, thin gut and fat attach.	
E46	"	4	19/03/07	F	21/03/07	Dead, milky white gut .	
E47	"	2	"	F	"	Dead , thin gut ,dark	
E48	"	4	"	M	"	Dead, thin brown gut with organic matter.	
E49	"	4	"	F	"	Dead, slightly swollen, milky gut.	+
E50	"	4	"	F	"	Live, swollen milky gut.	
